# Troponin T is elevated in a relevant proportion of patients with 5q-associated spinal muscular atrophy

**DOI:** 10.1038/s41598-024-57185-w

**Published:** 2024-03-19

**Authors:** Hanna Sophie Lapp, Maren Freigang, Johannes Friese, Sarah Bernsen, Victoria Tüngler, Maja von der Hagen, Patrick Weydt, René Günther

**Affiliations:** 1 Department of Neurology, University Hospital Carl Gustav Carus, Technische Universität Dresden, Dresden, Germany; 2 Department of Neuropediatrics, University Hospital Bonn, Bonn, Germany; 3 Department of Neurodegenerative Diseases, University Hospital Bonn, Bonn, Germany; 4 Department of Neuropediatrics, University Hospital Carl Gustav Carus, Technische Universität Dresden, Dresden, Germany; 5 German Center for Neurodegenerative Diseases, Dresden, Germany

**Keywords:** Motor neuron disease, Biomarkers

## Abstract

Troponin T concentration (TNT) is commonly considered a marker of myocardial damage. However, elevated concentrations have been demonstrated in numerous neuromuscular disorders, pointing to the skeletal muscle as a possible extracardiac origin. The aim of this study was to determine disease-related changes of TNT in 5q-associated spinal muscular atrophy (SMA) and to screen for its biomarker potential in SMA. We therefore included 48 pediatric and 45 adult SMA patients in this retrospective cross-sequential observational study. Fluid muscle integrity and cardiac markers were analyzed in the serum of treatment-naïve patients and subsequently under disease-modifying therapies. We found a TNT elevation in 61% of SMA patients but no elevation of the cardiospecific isoform Troponin I (TNI). TNT elevation was more pronounced in children and particularly infants with aggressive phenotypes. In adults, TNT correlated to muscle destruction and decreased under therapy only in the subgroup with elevated TNT at baseline. In conclusion, TNT was elevated in a relevant proportion of patients with SMA with emphasis in infants and more aggressive phenotypes. Normal TNI levels support a likely extracardiac origin. Although its stand-alone biomarker potential seems to be limited, exploring TNT in SMA underlines the investigation of skeletal muscle integrity markers.

## Introduction

5q-associated spinal muscular atrophy (SMA) is a rare neuromuscular disorder that is caused by loss-of-function mutations of the ubiquitously expressed *survival of motor neuron 1* gene (*SMN1, MIM* *600,354) and is clinically characterized by progressive, proximally pronounced muscle weakness and atrophy. Neuropathological studies revealed underlying degeneration of the motoneurons in the ventral horn of the spinal cord with secondary skeletal muscle atrophy and myopathy. However, developmental alterations of the postsynaptic motor endplate and neurodegeneration of other extra-motor tissues have been reported. SMA is classically divided into five subtypes according to the best ever reached motor milestone and the age at disease onset. Type zero, the congenital form, shows the most severe form of progression, while type four is relatively mild. While there were no treatment options until recently, the first disease-modifying therapy with nusinersen has been approved in 2016 by the FDA^1,2^. The stunning story of beneficial disease-modifying treatments for SMA has continued with the introduction of the *SMN2-*RNA splice modulator risdiplam^3,4^ and the *SMN1* gene replacement therapy onasemnogene abeparvovec-xioi^5^. While these disease-modifying therapies are targeting SMN depletion as the main contributor of the disease, more symptom-oriented approaches aiming to increase muscle strength and functional performance are under investigation. Fast skeletal muscle troponin activators lead to increased muscle strength relative to the neuronal input and are currently assessed in clinical trials^6,7^. Other approaches include myostatin inhibition^8^ or modulators of muscle metabolism^9^.

As there has no biomarker been validated of these new therapeutic options for the clinical routine yet, clinical scales remain the most relevant outcome parameter to date^10^. Major efforts for identifying suitable biomarkers have been undertaken but none have been implemented into clinical routine yet. Although muscle plays a key role in SMA pathology, research on fluid muscle biomarkers has been scarce. However, creatinine and creatine kinase have been discussed as candidate biomarkers in SMA, indicating that markers of muscle integrity are potentially useful for monitoring disease progression^11,12^.

Troponin I (TNI) and Troponin T concentration (TNT) are highly sensitive markers for myocardial ischemia^13^ with evidence for age- and sex-dependent upper reference limits of TNT^14,15^. However, elevated TNT but not TNI levels have recently been found in numerous systemic diseases, such as paraneoplastic systemic sclerosis^16^, inclusion body myositis^17^, polymyositis^18^ and other diseases involving skeletal myopathies^19,20^. An extracardiac origin of TNT^21^ and laboratory cross reactions with skeletal isoforms have been discussed^22,23^. Chronically elevated TNT levels were reported in amyotrophic lateral sclerosis (ALS)^24^, where TNT levels increased with disease duration and progression rate^25^. Furthermore, TNT levels correlated with motor function in patients with ALS. Of note, patients with the pure upper motoneuron variant primary lateral sclerosis (PLS) did not exhibit elevated TNT levels. Therefore, TNT was proposed as a biomarker of lower motoneuron or neuromuscular involvement^24^. Stevens et al. reported an altered expression of troponin isoforms and myosin heavy chains in infants with SMA type 1–3 (aged 1–5 months)^26^, while Djordjevic et al. did not find elevated TNT levels in children with SMA type 2 (mean age 5.4 years, range 2.5–11.7 years) and 3 (mean age 10.2 years, range 5.2–13.7 years)^27^.

This study aims to investigate disease-related changes of TNT and to screen for its biomarker potential in SMA.

## Material and methods

### Study design

The study was performed as a multicenter, retrospective, observational analysis. Inclusion criteria were the presence of 5q-associated SMA with molecular genetic proof of homozygous deletion or other mutation in the *SMN1* gene and the absence of a relevant cardiac disease based on the patients’ disease history. Data were collected from n = 45 children and n = 48 adults with SMA from the neurological and pediatric departments of the university hospitals in Bonn and Dresden (Germany). Informed consent was obtained from all subjects or their legal guardians. The conduct of the study was compliant with the World Medical Association Declaration of Helsinki and the guidelines and regulations of the respective local ethic committees, which approved the study (Bonn: Ethics Board decision letter 324/20, Dresden: EK 393,122,012 and EK 183,042,019).

### Laboratory analysis and functional scores

We retrospectively collected laboratory data prior to (treatment-naïve, baseline) and during treatment with disease-modifying treatments (nusinersen, risdiplam or onasemnogene abeparvovec-xioi) up to 38 months. According to the standard of care, nusinersen was administered intrathecally every four months after four initial loading doses. Risdiplam is a daily administered oral drug and onasemnogene abeparvovec-xioi is administered intravenously once, with the application being recommended before two years of age in Germany. The laboratory assays were performed at the in-house laboratory of the respective university hospital with standardized protocols used for clinical routine. Serum concentrations of TNT were analyzed in all samples using an electrochemiluminescence immunoassay (ECLIA) with an upper limit of normal of < 14 ng/L for all age and sex categories (Elecsys® Troponin T hs, pharmaceutical, Roche Diagnostics). Cardiac troponin I (TNI) was analyzed, whenever data were available, in order to screen for cardiac involvement (upper limit of normal: 10 ng/L). As TNI is more cardiospecific, TNT elevation without corresponding TNI elevation was postulated to originate from extracardiac tissue. Creatine kinase activity (CK, as a marker of muscle destruction, upper limit of normal: male 3.2 µkat/L, female 2.85 µkat/L^28^), myoglobin concentration (Myo, as a second marker of muscle destruction, upper limits of normal age- and gender-dependent, see^29^) and creatinine concentration (Crn, a surrogate marker of muscle mass, upper limits of normal age- and gender-dependent, see^30^) were included when available. Disease severity was assessed with the Revised Upper Limb Module (RULM)^31^, Hammersmith Functional Motor Scale Expanded (HFMSE)^32^ or the revised ALS-Functional Rating Scale (ALSFRS-R)^33^, which are commonly used scales to assess the motor skills and disease-related functional properties of patients with SMA.

### Statistical analysis

Statistical analysis and data visualization were performed using IBM SPSS Statistics Version 21.0. Unless otherwise stated, data are presented as mean ± standard deviation (SD). Shapiro–Wilk test was performed to test data on normal distribution and rank-based, non-parametric tests (Wilcoxon test, Mann–Whitney U test) were applied for non-normally distributed data. ANOVA was used additionally as it is insensitive of violations of normality in appropriate sample sizes^34^. The association between variables was analyzed rank-based using Spearman correlations and Eta^2^ for nominal variables. A correlation coefficient (ρ) of ρ < 0.3 was considered as a weak, ρ = 0.3–0.59 as a moderate, and ρ ≥ 0.6 as a strong correlation. An Eta^2^ (η^2^) of η^2^ = 0.01–0.059 was considered as weak, η^2^ 0.06–1.39 as moderate and ≥ 1.4 as strong effect^35^. Regressions were analyzed after adjustment for multicollinearity. TNT was entered as the dependent variable and those parameters that differed significantly between the groups with and without TNT elevation were entered as independent variables. Based on these results, further analyses were performed separately for adults and children. Longitudinal TNT data were examined using rank-based, non-parametric tests (Wilcoxon test). Data sets with missing values were excluded pairwise for cross-sequential analysis. Statistical significance was set as p < 0.05 two-sided.

## Results

In total, we included data from 93 patients with SMA of which 48 patients were adults (51.6%) and 45 were children below the age of 18 years (48.4%, see Table 1 and S1).

**Table 1:** Demographics of the study cohort.

	Adult cohort	Pediatric cohort
Sex	26 female, 22 male	20 female, 25 male
Age [years]	33.2 ± 12.5	3.3 ± 4.6
SMA type		Type 2: 12 (26.7%)
	Type 2: 17 (35.4%)	Type 1: 20 (44.4%)
	Type 3: 31 (64.4%)	Type 3: 6 (13.3%)
		NBS: 7 (15.6%)
Therapy	Nusinersen: 27 (56.3%)	Nusinersen: 14 (31.1%)
	Onasemnogene abeparvovec-xioi:	Onasemnogene abeparvovec-xioi: 31 (68.9%)
	Risdiplam: 19 (39.6%)	Risdiplam: 0
	None: 2 (4.2%)	None: 0
Study site	Dresden: 34 (70.8%)	Dresden: 10 (22.2%)
	Bonn: 14 (29.2%)	Bonn: 35 (77.8%)

NBS, diagnosed pre-symptomatically by newborn screening.

### Treatment-naïve troponin concentrations

#### TNT concentrations

Treatment-naïve TNT data were available for 77/93 patients (82.8%) with a TNT elevation in 47/77 patients (61.0%, see Table 2 and Fig. S1).

**Table 2:** Comparison between the group with and without baseline TNT elevation.

	Normal baseline TNT	Elevated baseline TNT	P
Sex	18 female, 12 male	20 female, 27 male	0.140
Age [years]	30.3 ± 15.5	13.6 ± 17.6	< 0.001
SMA type	Type 1: 1 (3.3%)	Type 1: 18 (38.3)	0.002
	Type 2: 12 (40.0%)	Type 2: 9 (19.1%)	
	Type 3: 15 (50.0%)	Type 3: 15 (31.9%)	
	NBS: 2 (6.7%)	NBS: 5 (10.6%)	
*SMN2* Copy Nr	2: 3 (10.0%)	2: 16 (34.0%)	0.048
	3: 21 (70.0%)	3: 22 (46.8%)	
	4: 6 (20.0%)	4: 9 (19.1%)	
TNT [ng/L]	8.91 ± 2.9	41.55 ± 29.9	< 0.001
BMI [kg/m^2^]	21.2 ± 6.1	17.4 ± 5.0	*
BMI (age- and sex-specific category)	Underweight: 42.3%	Underweight: 37.8%	0.69
	Normal weight: 38.5%	Normal weight: 40.0%	
	Overweight: 19.2%	Overweight: 22.2%	
CK [µkat/L]	1.79 ± 2.3	4.63 ± 8.4	< 0.001
Crn [µmol/L]	29.42 ± 10.4	25.87 ± 14.3	*
Crn (age- and sex-specific category)	Below LLN: 95.0%	Below LLN: 93.3%	0.84
	Normal: 5.0%	Normal: 6.7%	

*NBS* Newborn screening*, TNT* Troponin *T, BMI* Body mass index*, CK* = Creatine kinase activity*, Crn* Creatinine concentration, * just informational display, no comparison due to age- and sex-specific reference values (see below), *LLN* Lower limit of normal.

Post hoc tests revealed that SMA type 1 (55.75 ± 30.0 ng/L) had a significantly higher baseline TNT compared to type 2 (14.57 ± 8.5 ng/L; p < 0.001) or type 3 (17.94 ± 12.7 ng/L; p < 0.001), while TNT of SMA type 2 and 3 did not differ significantly (p = 0.46). These findings are consistent with lower *SMN2* copy numbers being moderately associated with higher baseline TNT (ρ = − 0.38, p < 0.001).

A regression analysis with TNT as dependent variable and age, SMA type, CK, Crn and BMI as independent variables revealed that age has a significant impact on TNT (standardized coefficient B = -0.61; p = 0.003). We therefore separated the cohort into adults and children for further analyses.

#### Adults

Treatment-naïve TNT concentrations were available for 41/48 adult patients with SMA (85.4%) with a treatment-naïve mean TNT of 14.34 ± 8.6 ng/L.

Elevated treatment-naïve TNT was found in 16/41 patients (33.3%) with a mean TNT of 23.11 ± 7.0 ng/L in the group with TNT elevation and 8.72 ± 2.9 ng/L in the group without TNT elevation.

Treatment-naïve TNT correlated strongly with CK (ρ = 0.65, p < 0.001) and moderately with Crn (ρ = 0.48, p = 0.008) while sex had a moderate effect (η^2^ = 0.30). There was no correlation with age (p = 0.57), BMI (p = 0.97), *SMN2* copy number (p = 0.43) or SMA type (p = 0.34). Although there was no correlation with SMA type, we analyzed group differences between SMA types 2 and 3 based on findings from Rudnik-Schöneborn et al.^36^ and Freigang et al.^11^ While there was no difference in TNT (SMA type 2: 12.8 ± 8.5 ng/L, SMA type 3: 15.2 ± 8.7 ng/L, p = 0.51), CK was higher in SMA type 3 (3.3 ± 3.5µkat/L) than SMA type 2 (1.2 ± 0.9 µkat/L, p = 0.033). Overall, CK was elevated in 21.1% of the adult patients.

Regression analysis in adult SMA including the above-mentioned variables revealed that sex had a significant impact on TNT (p = 0.003).

TNT was elevated in 3/20 (15.0%) female and 13/21 (61.9%) male patients. Males had significantly higher treatment-naïve TNT (male: 18.89 ± 9.2 ng/L, females: 9.56 ± 4.5 ng/L, p < 0.001) and CK (male: 3.68 ± 3.6 µkat/L, females: 1.71 ± 2.1 µkat/L, p = 0.001) compared to females. There was no difference between males and females in motor function (RULM: p = 0.52, HFMSE: p = 0.71), age (p = 0.21), SMA type (p = 0.47), *SMN2* copy number (p = 0.47), current (p = 0.35) or best ever motor milestone (p = 0.44), Crn (p = 0.15), or BMI (p = 0.98). There was also no difference in the distribution of age- and sex-specific BMI categories as per WHO classification (F(1, 42) = 1.09, p = 0.30) and respective Crn categories (F(1, 36) = 1.49, p = 0.23).

#### Children

Treatment-naïve TNT was available for 36/45 patients (80.0%) with a mean treatment-naïve TNT of 45.35 ± 33.6 ng/L at a mean age of 3.3 years.

TNT was elevated in 31/36 patients (86.1%) with a mean TNT of 9.86 ± 3.0 ng/L in patients without TNT elevation (mean age 4.7 years) and a mean TNT of 51.07 ± 32.7 ng/L in the group with TNT elevation (mean age 2.3 years). TNT was higher in children under the age of six months (11 children, mean age 1.6 months, mean treatment-naïve TNT 68.18 ng/L, TNT elevation in 81.8%) compared to children in the second year of life (Ten childrens, mean age 1.5 years, mean baseline TNT 29.01 ng/L, TNT elevation in 90%, p = 0.006) and children aged three years and older (10 children, mean age 8.5 years, mean treatment-naïve TNT 31.56 ng/L, TNT elevation in 70%; p = 0.029).

Treatment-naïve TNT correlated moderately and inversely with SMA type (ρ = -0.41, p = 0.014), *SMN2* copy number (ρ = -0.44, p = 0.008) and age (ρ = -0.43, p = 0.010) but not with BMI (p = 0.60), CK (p = 0.76) or Crn (p = 0.36). Sex had a very weak effect (η^2^ = 0.01). A regression analysis in children with SMA revealed no significant influence of any of the above-mentioned variables on TNT. Due to low data availability in some subtypes, reliable analyses of TNT and CK between SMA types were not possible in the pediatric cohort. CK was elevated in 25.0% of the children.

**Figure 1:**
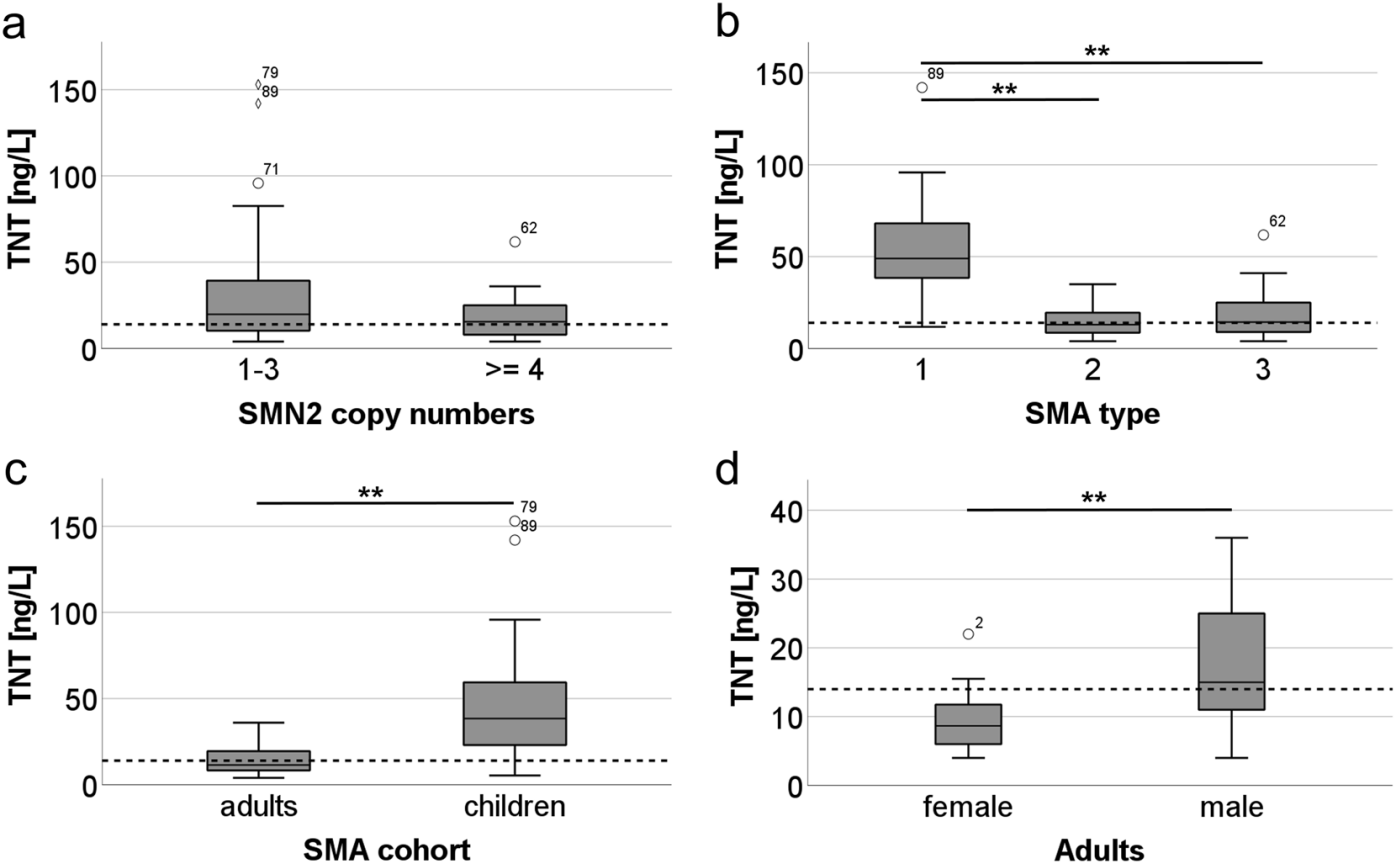
TNT treatment-naïve concentrations. A: TNT treatment-naïve concentrations per *SMN2* copy numbers, B: TNT treatment-naïve concentration between SMA types, C: TNT treatment-naïve concentrations between adults and children, D: TNT treatment-naïve concentration between male and female adult patients. Adult TNT cut-off (14 ng/L) displayed as dotted line. Whiskers indicate minimum and maximum, circles indicate extreme outliers with an interquartile range > 1.5 – 3 and diamond shapes indicate outliers with an interquartile range > 3. **p < 0.001. Patients identified by newborn screening are not displayed.

TNT was elevated in 17/18 (94.4%) female and 14/18 (77.8%) male patients and did not differ between both groups (male: 42.40 ± 33.4 ng/L, female: 48.29 ± 34.4 ng/L, p = 0.50; Fig. 1).

#### TNI concentrations

TNI data were available for 38/48 adult patients (79.2%) and 10/45 children (22.2%) with no TNI elevation in any patient.

### Dynamic of TNT concentrations under therapy

#### Adults

##### Nusinersen

**Figure 2:**
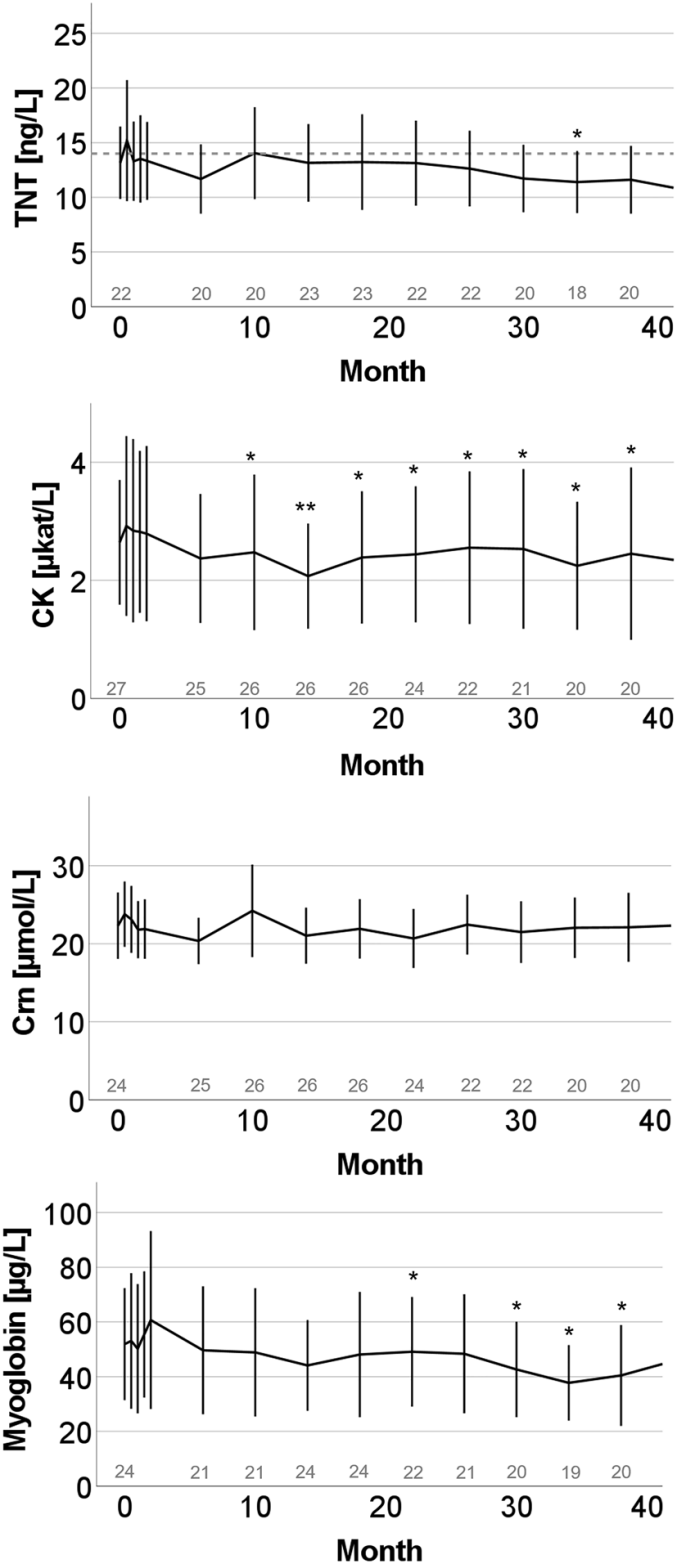
Longitudinal dynamic of TNT and skeletal muscle markers (CK, Crn, Myoglobin) in adult patients with SMA under nusinersen. Mean and standard deviation are displayed. TNT cut-off (14 ng/L) displayed as dotted line. Number of patients included in the analyses are indicated in gray. Significant changes from baseline are indicated by *p < 0.05 or **p < 0.001.

**Figure 3:**
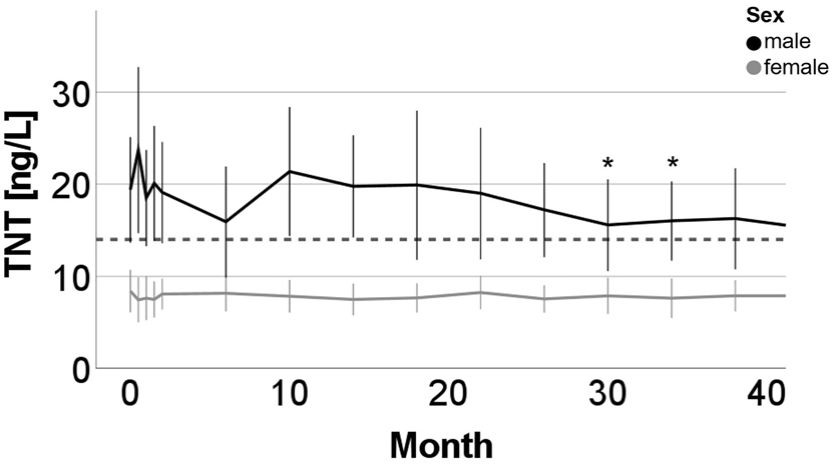
Longitudinal display of TNT in adult patients with SMA under nusinersen in sex subgroups. Mean and standard deviation are displayed. TNT cut-off (14 ng/L) displayed as dotted line. Significant changes from baseline are indexed in the respective color as follows: *p < 0.05.

27 adult patients with SMA (15 female; mean age 34.4 years, ranging from 18 to 56 years) received nusinersen. Treatment-naïve baseline TNT data were available for 22 patients (TNT: 14.4 ± 8.6 ng/L) with a TNT elevation in 9/22 patients (40.9%; 23.9 ± 7.4 ng/L). Except of significantly lowered TNT concentrations at 34 months (11.4 ± 6.1 ng/L, p = 0.038) compared to baseline, we did not observe significant changes on other timepoints (see Fig. 2). Subgroup analyses between male and female patients revealed a decrease from baseline TNT (20.6 ± 9.8 ng/L) in male patients after 34 months (16.0 ± 5.6 ng/L, p = 0.034) and 38 months (16.0 ± 6.1 ng/L, p = 0.049), but not in female patients (baseline: 8.6 ± 3.7 ng/L; 34 months: 7.6 ± 3.2 ng/L , p = 0.7; 38 months: 7.9 ± 2.4 ng/L, p = 0.4; see Fig. 3 and S2). Further analyses revealed a significant TNT decrease in the cohort with initial TNT elevation (baseline TNT: 23.9 ± 7.4 ng/L) after 34 months (TNT: 16.2 ± 6.2 ng/L, p = 0.028) and 38 months (TNT: 17.2 ± 6.6 ng/L, p = 0.028). There was no decrease in the cohort with normal baseline TNT (baseline: 8.2 ± 3.3 ng/L; 34 months: 8.1 ± 2.7 ng/L, p = 0.7; 38 months: 8.1 ± 2.6 ng/L, p = 0.4; see (Figs. S2 and S3).

CK was significantly decreased starting from month 10 till month 38 under therapy compared to baseline (baseline: 2.9 ± 3.1 µkat/L, 10 months: 2.6 ± 3.4 µkat/L (p = 0.04), 30 months: 2.5 ± 2.9 µkat/L (p = 0.003)). Crn did not show a relevant change compared to baseline at any point during therapy (baseline: 22.2 ± 11.8 µmol/L, 2 months: 22.3 ± 9.4 µmol/L, 10 months: 24.4 ± 15.2 µmol/L, 30 months: 21.5 ± 8.4 µmol/L). Myoglobin was significantly decreased compared to baseline (58.1 ± 60.0 µg/L) at 22 months (48.4 ± 46.5 µg/L, p = 0.03) and consistently after 30 months of therapy (37.8 ± 29.5 µg/L, p = 0.013) (see Fig. 2).

##### Risdiplam

19 adult patients with SMA (9 female; mean age 31.9 years, ranging from 18 to 58 years) received risdiplam. Baseline TNT data were available for 17 patients (TNT: 13.8 ± 7.9 ng/L) with a TNT elevation in 6/17 patients (35.3%, TNT: 22.1 ± 7.6 ng/L). Only a short observation period is available, which leads to strong limitations to draw robust conclusions on the following results. In this limited data set, TNT did not change under risdiplam, neither after two months (TNT: 13.4 ± 7.9 ng/L, p = 0.17) nor after six months (TNT: 12.1 ± 4.6 ng/L, p = 0.60, see Fig. S4). Subgroup analyses revealed decreased TNT concentrations in the group with initial TNT elevation after two months (5 patients, TNT: 18.4 ± 8.6, p = 0.042). Later analyses were hindered by data availability and available data sets for only 2 patients after 6 months of treatment. There were no changes from baseline in the group with normal baseline TNT and no changes in any sex subgroup.

##### Onasemnogene abeparvovec-xioi

No data of onasemnogene abeparvovec-xioi-treated adults were available.

#### Children

##### Nusinersen

14 pediatric patients with SMA (Six female; mean age 8.2 years, ranging from 3 months to 15.8 years) received nusinersen. Baseline TNT data were available for only six patients (TNT: 39.8 ± 18.3 ng/L) with a TNT elevation in 5/6 patients (83.3%, 45.1 ± 14.3 ng/L). After 18 months of treatment, TNT data were available for only three patients. Due to the limited number of patients, analysis was done on a descriptive basis only (Fig. S5).

##### Risdiplam

No data of risdiplam-treated children were available.

##### Onasemnogene abeparvovec-xioi

31 pediatric patients (14 female; mean age 14 months, ranging from newborns to 2 years) received onasemnogene abeparvovec-xioi. Baseline TNT data were available for 30 patients (46.5 ± 36.0 ng/L) with a TNT elevation in 26/30 patients (86.7%, TNT: 52.2 ± 35.3 ng/L). Only a short observation period is available, which leads to strong limitations to draw robust conclusions on the following results. In this limited data set, TNT did not change under onasemnogene abeparvovec-xioi, neither after 2 months (TNT: 44.8 ± 36.0 ng/L, p = 0.84) nor after 6 months (TNT: 54.8 ± 38.5 ng/L, p = 0.12, see Fig. S6). Subgroup analyses of the cohort with initial TNT elevation revealed no changes up to 6 months of treatment (TNT: 59.7 ± 25.4 ng/L, p = 0.17).

## Discussion

We found elevated concentrations of TNT in more than half of treatment-naïve patients with SMA in our cohort, a marker primarily used for myocardial infarction. In contrast, none of the samples studied had concentrations of the myocardial specific TNI above the cut-off values. This strongly argues against myocardial damage and we therefore speculate that TNT originates from degenerating or regenerating skeletal muscle fibers in patients with SMA. Animal studies have demonstrated that cardiac TNT is physiologically expressed in the rat skeletal muscle throughout embryonic and fetal development, disappears during the first weeks after birth and is re-expressed in regenerating muscle fibers after an injury or after degeneration^37^. Similarly, TNT but not TNI were expressed in muscles of patients with other neuromuscular disorders such as Duchenne muscular dystrophy^21,38^, and TNI was not expressed in fetal and healthy or diseased adult skeletal muscle^39^.

Existing literature indicates that TNT levels are higher in healthy neonates and gradually decrease with age, largely during the first 3 months of life^40^. TNT values above the cut-off of 14 ng/L in healthy children were previously reported, particularly up to the age of 6 months^14^. The highest TNT levels measured up to 37 ng/L (97.5th percentile) and declined with age. At one year of age, the 97.5th percentile dropped to the adult cut-off of 14 ng/L. As a result, the upper limit of normal of the assay we used does not seem to be suitable for young children. We therefore compared our data with the reference values developed by Kiess et al.: In 86% of the treatment-naïve infants with SMA and in 90% of children in the second year of life in our cohort, we found TNT concentrations above this 97.5th percentile of healthy (compare Fig. S1). TNT elevation was more than twice as prominent and three times higher in children compared to adults. Furthermore, treatment-naïve TNT concentrations in children correlated inversely with the SMA type, *SMN2* copy number and age, suggesting an association of TNT concentration with more aggressive subtypes in combination with early disease stages^41^. During aging, restoration of muscle maturation and nerve function following injuries are delayed^42,43^ and non-neurodegenerative processes such as inflammation and secondary myopathy might outweigh motoneuron loss in later stages^44^. Thus, more active denervation and regeneration processes in muscles of the aggressive phenotype SMA type 1 might result in a stronger re-expression of TNT in the skeletal muscles compared to milder phenotypes. Still, treatment-naïve TNT was above the established laboratory cut-off in approximately one third of the adult patients with SMA. Here, TNT concentrations correlated with the muscle destruction marker CK and the muscle mass marker Crn, supporting the hypothesis of a skeletal muscle origin of elevated TNT concentrations in SMA^11^. Our findings on CK in adult SMA patients are concordant with findings from Rudnik-Schöneborn et al. and Freigang et al., who found elevated CK concentrations in milder SMA types^11,36^. Possible explanations include secondary myopathic processes in patients with more remaining vulnerable muscle mass, leading to increased absolute CK levels in the peripheral blood. As most of our SMA patients show strong muscle atrophy, the standard absolute CK reference values might not be suitable for these patients. Whether or not patients with severe muscle atrophy show relatively increased CK must be studied under consideration of the lean body mass in future studies.

A second striking result was a clear sex difference in TNT elevation in adult patients with SMA, which seems to be much more pronounced than recent cardiologic studies reported for sex differences between healthy males and females^45–47^. Although we did not observe differences in motor function, male patients showed significantly higher baseline TNT levels than females. We suspected an effect of muscle mass, however, TNT correlated only moderately to Crn but not to BMI in our adult cohort and there were no significant differences of Crn or BMI between adult male and female patients. However, further analyses including quantitative analyses like dual x-ray absorptiometry or other assessments of lean body mass are warranted to exclude an impact of muscle mass on sex-dependent TNT concentrations in patients with SMA. A second hypothesis was an association with sex hormones. Several studies reported a TNT decrease under testosterone therapy^48,49^, making it rather unlikely that the sex hormone testosterone is responsible for the TNT difference between male and female patients. At present, we do not have a conclusive explanation for the sex difference in our cohort.

Longitudinal analysis of TNT concentrations during disease-modifying treatment with nusinersen in adult patients with SMA revealed a significant decrease after 34 and 38 months of treatment in the subcohort of patients with elevated TNT concentrations at baseline. Quantitative magnetic resonance imaging showed stabilized muscle mass under nusinersen treatment, indicating a slowed or reduced muscle wasting. Such reduced muscle wasting under nusinersen might result in decreasing CK and TNT levels (compare^11,50^). Compared to the dynamics of the muscle integrity markers CK and myoglobin during treatment in our study, the aforementioned dynamic of TNT seems to be much weaker.

No sufficient data for adults treated with risdiplam or children and infants treated with risdiplam and nusinersen were available. In the group of onasemnogene abeparvovec-xioi-treated infants and children with SMA, no significant decrease of TNT concentration was observed in the very short observation period of 6 months. We did not observe a TNT or TNI increase shortly after administration of onasemnogene abeparvovec-xioi, suggesting that none of our patients experienced a drug-related myocarditis, a feared side effect of the treatment. Longer observation periods would be needed to draw proper conclusions on TNT dynamics in infants and children during disease-modifying treatment.

In conclusion, we observed an extracardiac TNT elevation in a relevant proportion of patients with SMA, supporting the potential usefulness of fluid molecular skeletal muscle markers as biomarkers for SMA. Interestingly. TNT was more pronounced in more aggressive subtypes in early disease stages. We hypothesized that possible mechanisms might include differential effects in children and adults: While TNT elevation in children might result from more active denervating and regeneration processes with altered TNT expression in the muscle, it might stem from muscle destruction processes in progressed disease stages. Nonetheless, this hypothesis is highly speculative and further research is warranted. Taking into account the limited longitudinal dataset, the dynamic of TNT during the disease-modifying treatment nusinersen showed a slight decline in the subset of patients with initially elevated TNT levels in adults with SMA. However, in comparison to CK or Myoglobin, the added biomarker value of TNT for monitoring therapeutic effects under disease-modifying therapies seems to be low. Nevertheless, our data argue for further investigations on molecular biomarkers of the skeletal muscle in patients with SMA.

Our study has limitations. It is a retrospective cross-sequential observational study and as such, there were numerous missing data points. The cohorts for subgroup analyses were relatively small and the observation periods relatively short, which further limited data analysis, particularly in infants and children. Further, as lined out above, CK analyses and Crn analyses with the Jaffe method showed a relevant floor effect that limited our data analysis. Investigating lean body mass could have added valuable information but was not collected. Data were collected in two different centers in Germany and reference values and units differed for some variables. Finally, we are aware that we operated with cut-off values for TNT and TNI that were optimized to detect cardiac events. Although TNI levels below the threshold of detection have a high negative predictive value for cardiac diseases^51^, it must be noted that TNI alone cannot rule out underlying cardiac disease. However, given the low pretest probability in our cohort due to application of the exclusion criteria, relevant cardiac diseases seem unlikely. Future research will show whether monitoring of neuromuscular processes requires different cut-off values than cardiac events.

### Supplementary Information

Supplementary Information.

## Data Availability

Due to regulations of the ethics committee, the full data cannot be made available publicly. However, data access will be provided to other researchers upon request. In this case, please contact the corresponding author (rene.guenther@uniklinikum-dresden.de).
