# Supplementary material for: Troponin T is elevated in a relevant proportion of patients with 5q-associated spinal muscular atrophy
Source: Sci Rep. 2024 Mar 19;14:6634. doi: 10.1038/s41598-024-57185-w (PMC10951305; doi:10.1038/s41598-024-57185-w)
Supplement: Supplementary file 1 — Supplementary Information. [file 41598_2024_57185_MOESM1_ESM.pdf]

Troponin T is elevated in a relevant proportion of patients with 5q-associated spinal muscular atrophy

Hanna Sophie Lapp, MD [1]; Maren Freigang [1]; Johannes Friese, MD [2]; Sarah Bernsen, MD [3]; Victoria Tüngler, MD [4]; Maja von der Hagen, MD [4]; Patrick Weydt, MD [3]; René Günther, MD [1,5*]

1 Department of Neurology, University Hospital Carl Gustav Carus, Technische Universität Dresden, Dresden, Germany
2 Department of Neuropediatrics, University Hospital Bonn, Bonn, Germany
3 Department of Neurodegenerative Diseases, University Hospital Bonn, Bonn, Germany
4 Department of Neuropediatrics, University Hospital Carl Gustav Carus, Technische Universität Dresden, Dresden, Germany
5 German Center for Neurodegenerative Diseases, Dresden, Germany

* *Corresponding author:* René Günther, Department of Neurology, University Hospital Carl Gustav Carus, Fetscherstraße 74, 01307 Dresden, E-Mail: rene.guenther@uniklinikum-dresden.de

Tab. S1: Correlation between demographic variables of the study population

		SMA type	SMN2 copy nr	age	BMI
**SMA type**	*correlation coefficient ρ*	1.000	$0.45^{\#}$	$0.39^{\#\#}$	$0.32^{\#}$
	p		<0.001	<0.001	0.004
***SMN2* copy nr**	*correlation coefficient ρ*	$0.45^{\#}$	1.000	$0.49^{\#}$	$0.45^{\#}$
	p	<0.001		<0.001	<0.001
**Age**	*correlation coefficient ρ*	$0.39^{\#}$	$0.49^{\#}$	1.000	$0.65^{\#\#}$
	p	<0.001	<0.001		<0.001
**BMI**	*correlation coefficient ρ*	$0.32^{\#}$	$0.45^{\#}$	$0.65^{\#\#}$	1.000
	p	0.004	<0.001	<0.001	
**Sex**	*η^2*	0.08^{++}	0.03^{+}	0.09^{++}	0.00

Note: *ρ = Spearman-Rho, $^{\#}$ = moderate correlation, $^{\#\#}$ = strong correlation, η^2 = Eta², $^{+}$ = weak effect, $^{++}$ = moderate effect, BMI = Body mass index*

Fig.S1

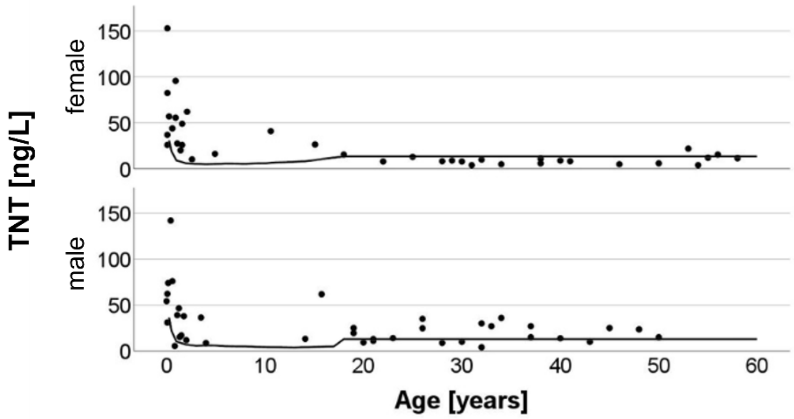

Treatment-naïve TNT per sex. *Individual data. Line indicates the 97.5th percentile of TNT in healthy children per age group*

Fig.S2

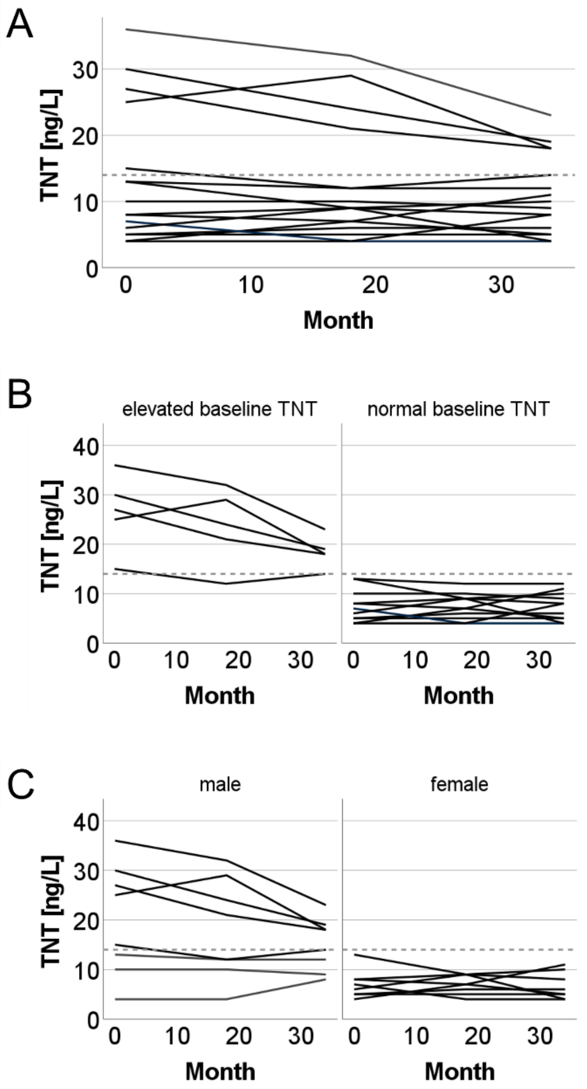

Longitudinal data of intraindividual TNT in adult patients with SMA under nusinersen*. A: total cohort, B: groups stratified by baseline TNT, C: groups stratified by sex. TNT cut-off (14 ng/L) displayed as dotted line*

Fig.S3

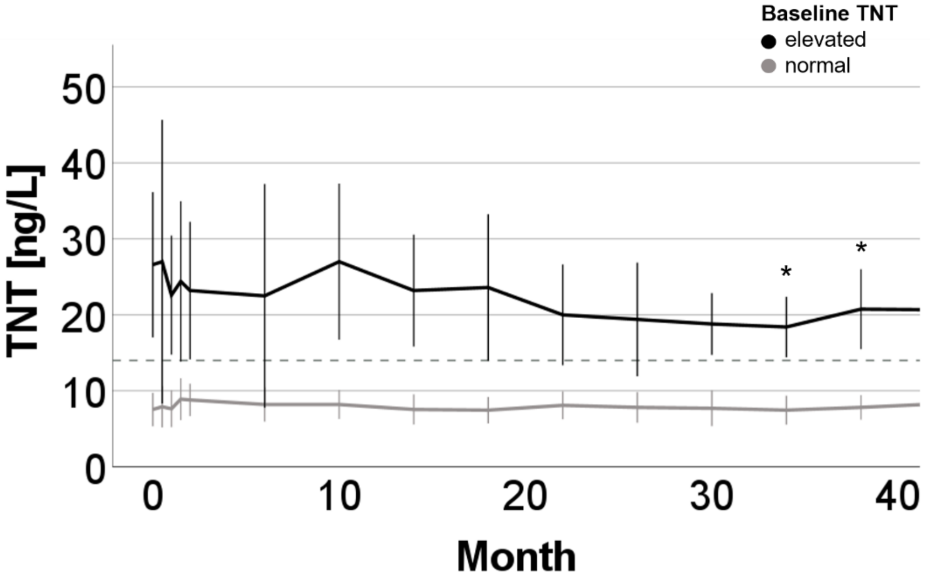

Longitudinal data of TNT in adult patients with SMA under nusinersen stratified by baseline TNT. *Mean and standard deviation displayed. TNT cut-off (14 ng/L) displayed as dotted line. Significant changes from baseline are indexed as follows: * = $p < 0.05$*

Fig.S4

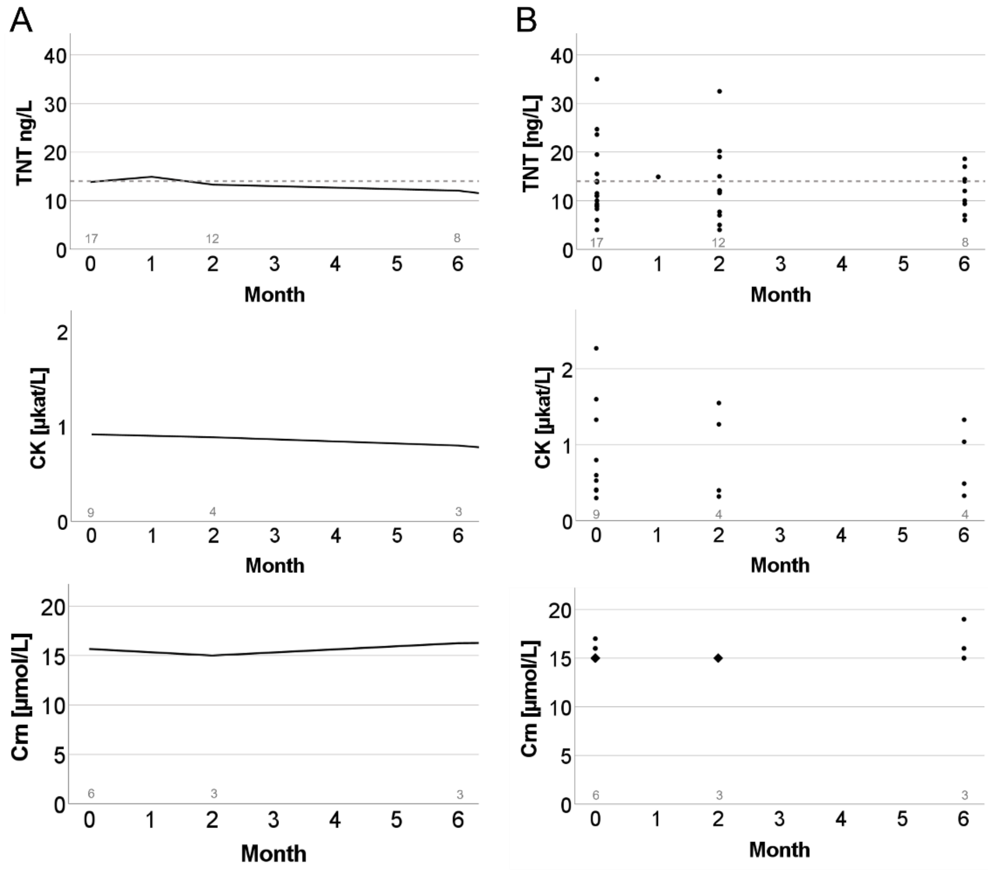

Longitudinal data of TNT and skeletal muscle markers (CK, Crn) in adult patients with SMA under risdiplam. *A: Mean concentration, B: individual value plot. ◆ Indicate more than one data point. TNT cut-off (14 ng/L) displayed as dotted line. Number of patients included in the analyses are indicated in gray.*

Fig.S5

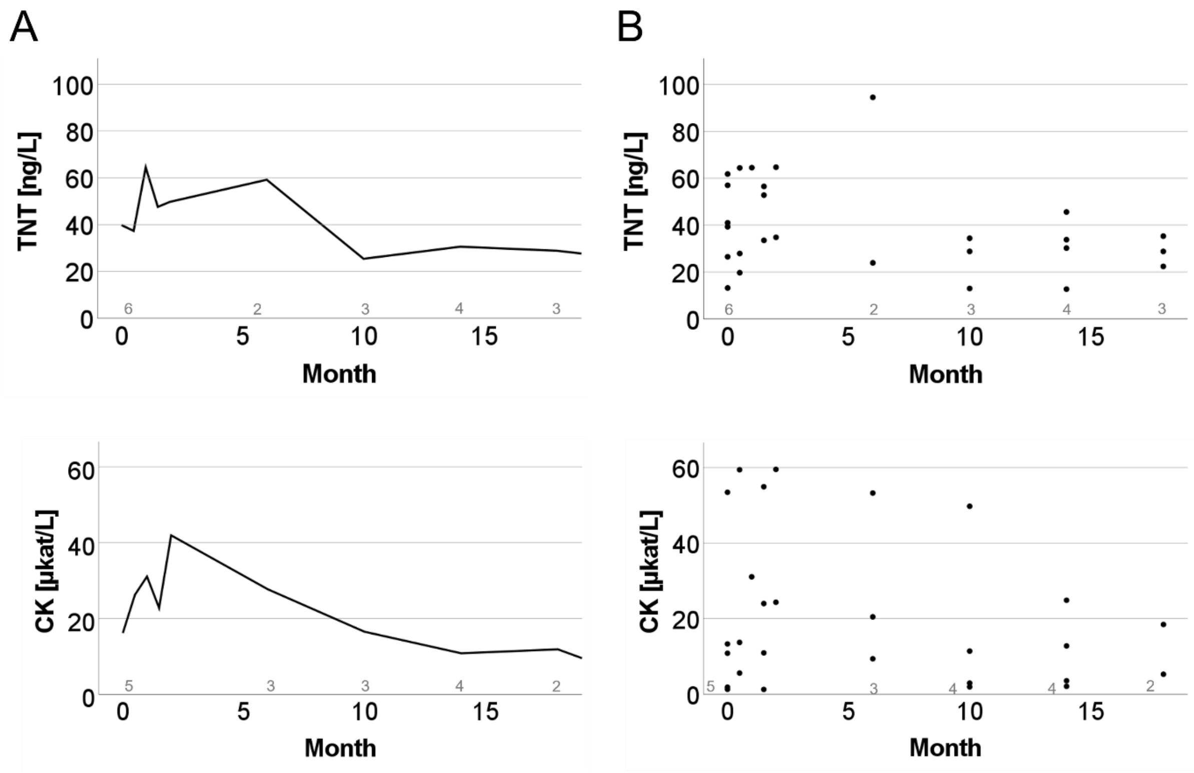

Longitudinal data of TNT and CK in pediatric patients with SMA under nusinersen. *A: Mean concentration, B: Individual value plot. TNT cut-off (14 ng/L) displayed as dotted line. Number of patients included in the analyses are indicated in gray*

Fig.S6

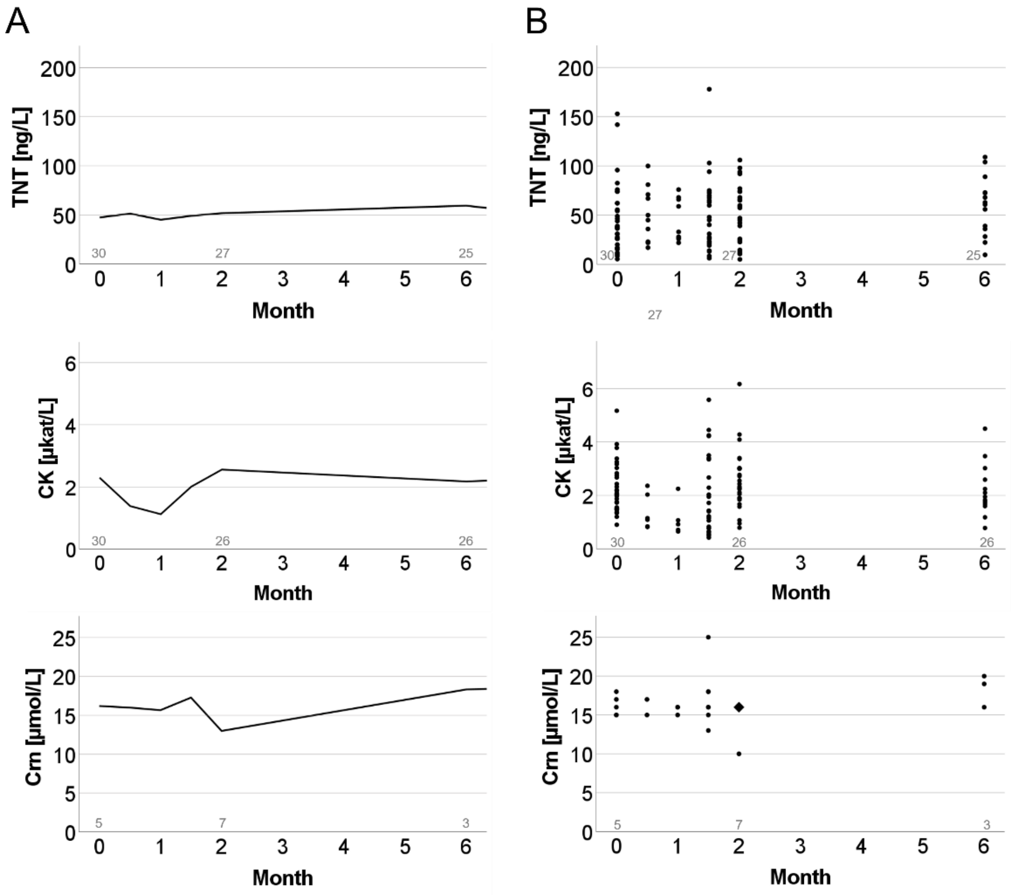

Longitudinal data of TNT and skeletal muscle markers (CK, Crn) in pediatric patients with SMA under onasemnogene abeparvovec-xioi. *A: Mean concentration, B: Individual value plot. ◆ Indicate more than one data point. TNT cut-off (14 ng/L) displayed as dotted line. Number of patients included in the analyses are indicated in gray.*
